# Trio fluorophore-based phenotypic assay for the detection of artemisinin-induced growth-arrested *Plasmodium falciparum* in human erythrocytes

**DOI:** 10.1038/s41598-024-52414-8

**Published:** 2024-01-20

**Authors:** Porntida Kobpornchai, Mallika Imwong, Kasem Kulkeaw

**Affiliations:** 1 Siriraj Integrative Center for Neglected Parasitic Diseases, Department of Parasitology, Faculty of Medicine Siriraj Hospital, Mahidol University, Bangkok, 10700 Thailand; 2 Siriraj-Long Read Lab, Department of Bioinformatics, Faculty of Medicine Siriraj Hospital, Mahidol University, Bangkok, 10700 Thailand; 3 Department of Molecular Tropical Medicine and Genetics, Faculty of Tropical Medicine, Mahidol University, Bangkok, 10700 Thailand

**Keywords:** Biological techniques, Parasitology, Infectious diseases, Medical research

## Abstract

Artemisinin combination therapy remains effective for the treatment of falciparum malaria. However, *Plasmodium falciparum* can escape the effects of artemisinin by arresting their growth. The growth-arrested parasites cannot be distinguished from nonviable parasites with standard microscopy techniques due to their morphological similarities. Here, we demonstrated the efficacy of a new laboratory assay that is compatible with the artemisinin susceptibility test. As a result of the differential cell permeabilities of two DNA-binding fluorophores, growth-arrested *P. falciparum* can be distinguished from parasites killed by artemisinin, since the latter lose cell membrane permeability. This fluorescence-based assay increased the sensitivity and specificity of the ring survival assay in the assessment of artemisinin susceptibility. When combined with a third fluorophore-conjugated anti-human leukocyte antibody, this trio fluorophore assay became more useful in identifying growth-arrested parasites in mock human blood samples. This novel assay is a simple and rapid technique for monitoring artemisinin resistance with greater sensitivity and accuracy compared with morphology-based observations under a light microscope.

## Introduction

In the last decade, several countries have put great effort into eradicating malaria, contributing to a substantial reduction in global mortality rates. Despite this effort, malaria caused by *Plasmodium falciparum* remains a great public health concern and ranks in the top 5 of the world’s deadliest infections. Since 2000, the number of infected individuals per year has been almost constant, affecting more than 200 million people. In 2021, over 600,000 malaria deaths were documented by the World Health Organization^1^. Historically, the drug resistance of *P. falciparum* has always posed a major threat and challenged efforts to eradicate malaria. High biological adaptability allows the parasite to evade the effects of many antimalarial regimens^2^. To stop the spread of drug-resistant parasites, a surveillance system with tools to test for drug susceptibility and resistance remains essential.

Artemisinin combination therapies (ACTs) have been the first-line treatments for uncomplicated falciparum malaria since 2006*.* Artemisinin (ART) and its derivatives are regarded as fast-acting drugs because they are capable of reducing the number of *P. falciparum* parasites are in the asexual intraerythrocytic development cycle during the first 3 days of treatment. To ensure a robust treatment response, a long-acting drug (e.g., lumefantrine, piperaquine and mefloquine) is administered in combination therapy to eliminate the residual parasites^3,4^. Despite its high potency, recent reports have shown an increase in artemisinin resistance-associated mutations in the main malaria endemic areas, including the Greater Mekong subregion (GMS)^5^ and some parts of Africa^6^. As a clinical form of ART resistance, artemisinin clears parasites from the blood at a slower rate after monotherapy. A proposed mechanism underlying the tolerance to artemisinin treatment is the growth arrest of the quiescent forms of the parasites, in which artemisinin-exposed parasites halt metabolism and stop their growth and proliferation. Several days after artemisinin exposure, parasites in the temporary ring stage growth arrest form resume a normal development cycle, leading to a second clinical phase called recrudescence^7^. The potential drug resistance of the arrested *P. falciparum* remains unproven. Nevertheless, these circumstances may lead to the spread of drug-resistant parasites. Thus, a surveillance system for monitoring and testing the drug resistance of *P. falciparum* is essential in both clinical and field settings.

Morphological examination of Giemsa-stained blood smears is a standard microscopic method commonly used in the malaria diagnosis, *Plasmodium* species identification^8^ and the ring-stage survival assay (RSA) phenotype^9^. The morphology of growth- arrested parasites is similar to that of the pyknotic form of dead parasites, which have condensed chromatin and scant cytoplasm^10^. Therefore, growth-arrested and pyknotic parasites cannot be distinguished through microscopic observation, and there is a 19.4% rate of false negative results^11^. The skill and experience of the examiners are part of the problem; however, the microscopic method is also laborious, time-consuming and subjective. Interpretations of growth arrest and pyknosis vary among examiners. Other in vitro assays of drug susceptibility primarily rely on measuring metabolism or byproducts such as hemozoin^12^. However, these assays are not applicable for identifying growth-arrested *P. falciparum* due to a lack of these phenotypes.

The use of fluorescence probes (fluorophores) in flow cytometry and high-content imaging facilitate qualitative and quantitative cell analyses. Fluorescent signals have high sensitivity and specificity when combining fluorophores that specifically tag different targets. Several commercially available fluorophores bind to specific compartments of malaria parasites, including the nucleus, mitochondria, the endoplasmic reticulum and the food vacuole. In peripheral blood, mature erythrocytes are enucleated cells, which allows the specific detection of intraerythrocytic *P. falciparum* using DNA-binding fluorophores^13^. Many nucleic acid-binding fluorophores are extensively used for the analysis of parasite invasion and growth, drug screening, and diagnosis. These fluorophores are classified based on cell permeability. Cell permeant fluorophores include SYBR Green I^14^, YOYO-1^15^, Hoechst 33258^16^, and Hoechst 33342^17^ and cell impermeant fluorophores include hydroethidine^18^, ethidium bromide^19^, and propidium iodide^20^. ViSafe Green (VSG) is a cell-permeable fluorophore that was originally used for visualizing separated DNA or RNA on agarose or polyacrylamide gels. Our group demonstrated that VSG could be used to assess the development of *P. falciparum* in vitro^21^. However, one significant disadvantage of single-use DNA fluorochromes is that they cannot differentiate between viable and dead parasites. In this study, we reasoned that the artemisinin-induced death of *P. falciparum* results in the loss of parasite membrane integrity. Thus, growth-arrested and dead parasites could be distinguished based on the cell permeability of the fluorophore. Due to the selective properties of the erythrocyte membrane, a mild detergent is used to enhance erythrocyte entry. Moreover, we developed a trio fluorophore in a single tube for the discrimination of growth-arrested and dead *P. falciparum*. This assay can be used as an artemisinin susceptibility test in both in vitro and ex vivo settings.

## Results

### DHA induces growth-arrest in ring-stage P. falciparum strain K1

**Figure 1:**
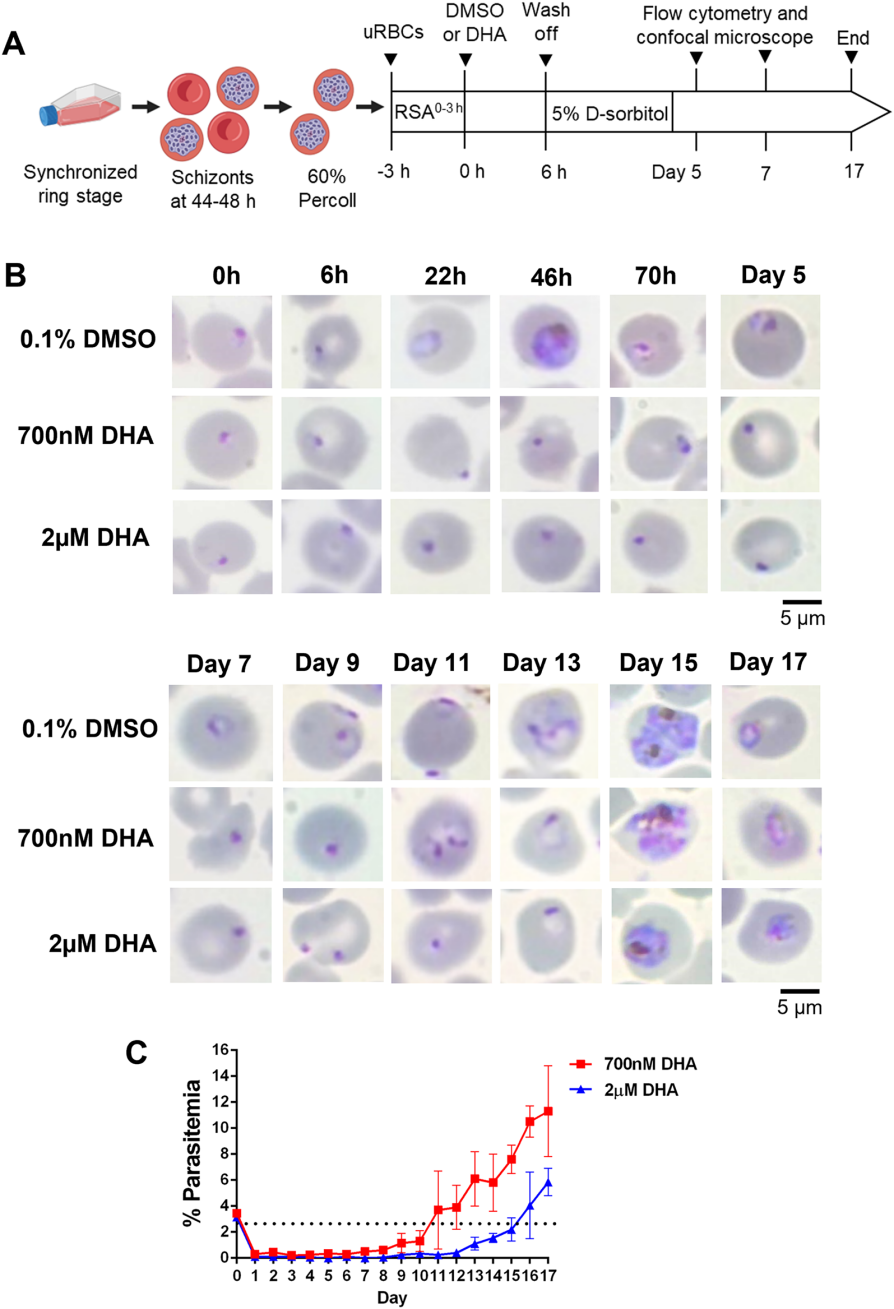
Induction of intraerythrocytic dormancy in *P. falciparum* strain K1 after DHA treatment. (**A**) Schematic diagram of the in vitro ring-stage survival assay (RSA). The parasites were synchronized through two rounds of sorbitol treatments followed by an additional culture for 44–48 h to obtain schizonts. Sixty percent Percoll was used to enrich schizonts before culture with uninfected erythrocytes. Early ring-stage trophozoites were obtained within 3 h of culture. At the start of the DHA experiment (t = 0 h), the ring-stage trophozoites at 3% parasitemia were exposed to either 700 nM DHA, 2 µM DHA or 0.1% DMSO for 6 h, washed, and returned to the culture. Parasites exposed to DHA or DMSO were treated with 5% sorbitol to remove the mature stage every 24 h for three days. On days 5 and 11, 1 mL of each culture was subjected to enrichment of the intraerythrocytic parasites by incubation with streptolysin O and subsequent gradient centrifugation in Percoll (method known as SLOPE). Live and dead parasites were verified using flow cytometry and confocal microscopy. The recrudescence of parasites was monitored daily by using Giemsa-based microscopy for up to 17 days. (**B**) Representative microscopic images of Giemsa-stained erythrocytes on thin blood smears. Scale bar = 5 µm. (**C**) Recrudescence curve of *P. falciparum* strain K1 exposed to 700 nM (red line) and 2 µM (blue line) DHA. Parasitemia was examined by two independent readers blinded to the slide identifications. Percentages of parasitemia are presented as the mean ± SEM of three independent experiments. The dotted line indicates 1% parasitemia, the limit of detection by the standard microscope.

Previous in vitro experiments showed that temporal growth-arrested *P. falciparum* could be readily induced by DHA^9^. To further expand on this observation, we monitored the growth of synchronized ring-stage parasites after 6 h of exposure to DHA (Fig. 1A). Three successive 5% D-sorbitol treatments at 24, 48, and 72 h excluded the nonring stage parasites that were not affected by DHA or those that had recovered from growth arrest within the first 72 h of DHA exposure (Fig. 1A). Parasite growth was monitored daily using Giemsa-based microscopy (Fig. 1B). After 6 h of treatment, the DMSO- and DHA-exposed parasites presented the same morphology, in which the diameter ratio of the nucleus and cytoplasm remained unchanged when compared to that stage at pretreatment (0 h). At 22 h postculture of the DMSO-treated parasites, the ring-stage parasites developed into growing trophozoites and mature schizonts, while the DHA-treated parasites had condensed nuclei and reduced cytoplasm, which are characteristic of pyknotic-like cells (Fig. 1B). The intraerythrocytic stage and number of parasitized cells were monitored to observe recrudescence. At the initial drug exposure, 3% of the ring-stage parasites were treated with 700 nM or 2 µM DHA. After 6 h of culture of the DHA-exposed parasites, parasitemia decreased to a level lower than 0.1%. From day 11 onward, the percentage of parasitemia increased by more than 3% following treatment with 700 µM DHA (red line, Fig. 1C). In contrast, the parasites exposed to 2 µM DHA took up to 16 days to recovery more than 3% parasitemia. At day 17 of the culture, parasitemia rose to 11.3% in the 700 nM DHA-treated parasites. However, only 5.8% parasitemia was measured after treatment with 2 µM DHA. Thus, higher concentrations of DHA delayed the recrudescence of the parasites. To evaluate if the stress arising might be caused by the synchronization protocol, the DMSO or DHA- parasite erythrocytes were treated three times with or without 5% D-sorbitol every 24 h beginning at early ring stage at 0–3 h post-merozoite invasion. The results showed that sorbitol treatment had no impact on the growth curve of DHA-treated parasites (see Supplementary Figure S1 online). These findings confirm that in vitro exposure to DHA induces growth arrest in *P. falciparum*, which is morphologically indistinguishable from pyknotic parasites. However, growth-arrested parasites can reenter the cycle of intraerythrocytic growth.

### Triton X-100-containing buffer does not impair parasite growth

**Figure 2:**
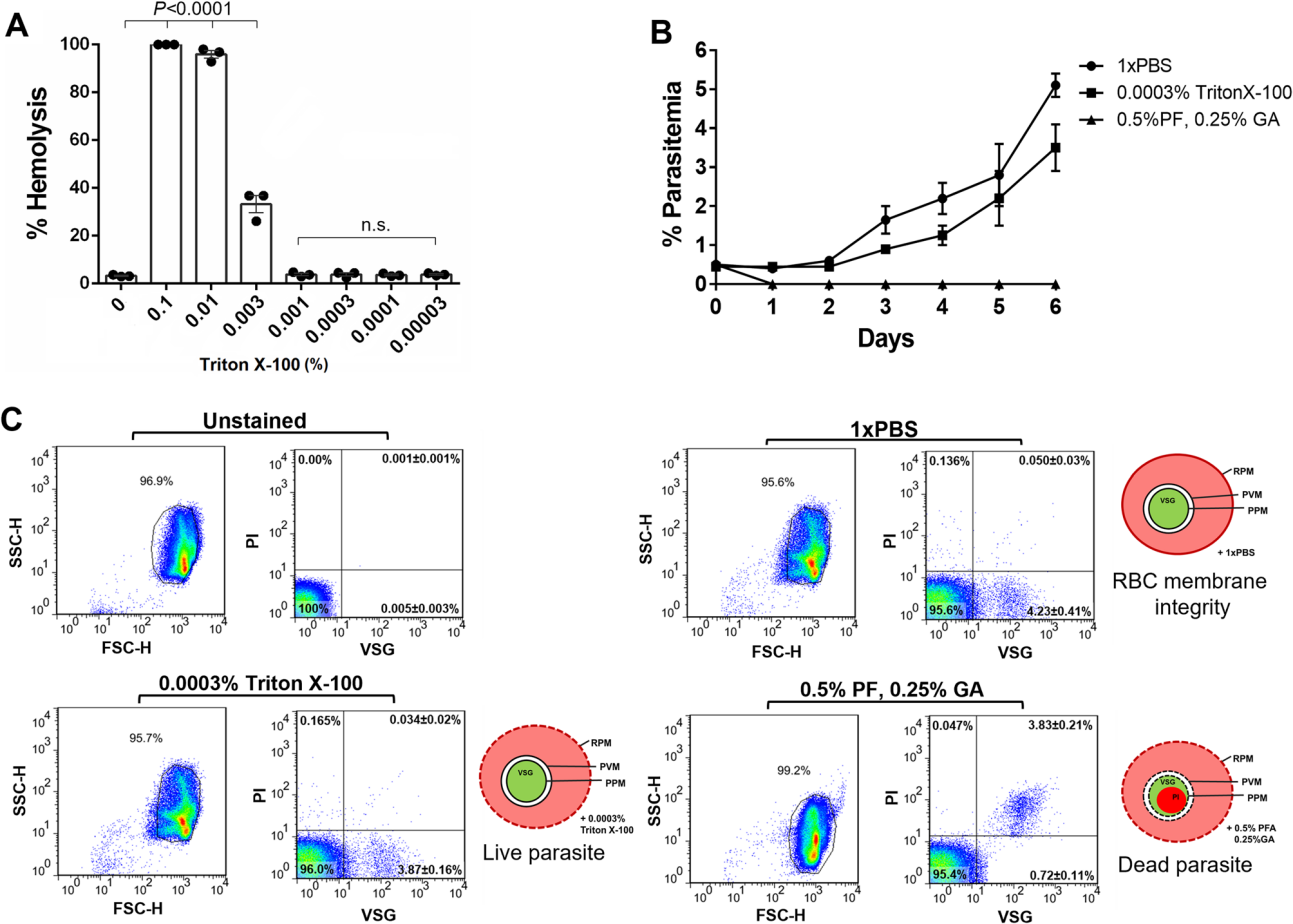
Effect of Triton X-100 on the viability of *P. falciparum* stain K1. (**A**) Percentage of hemolysis of the uninfected erythrocytes was dependent on the concentration of Triton X-100. Relative to the background control (0% Triton X-100), Triton X-100 at a concentration of 0.0003% was the minimal hemolysis concentration. (**B**) Recovery of ring-stage *P. falciparum* after exposure to cell membrane-permeating reagents. Initial 0.5% ring-stage parasites were exposed to 0.0003% Triton X-100 or 0.5% PFA/0.25% GA. After removing the detergents, parasite growth was monitored for 6 days. PBS was used as a negative control. Parasitemia was calculated using Giemsa-based microscopy. (**C**) Dot plot of the representative flow cytometry analysis of the parasites at day 6 post culture. The parasites were stained with 10 µg/mL VSG and 10 µg/mL PI before analyzing the fluorescent signal. Cells were gated based on the high intensity of FSC and SSC followed by profiling according to the fluorescence intensity of PI and VSG. Live parasites were positive for VSG but negative for PI. Due to loss of permeability of the parasite membrane, the dead parasites were positive for both VSG and PI. A cartoon depiction of live and dead parasites is shown. The RBC plasma membrane (RPM) was permeated using the 0.0003% TritonX-100 (dotted line of red circle). Parasites are shown in green due to the emitted fluorescence signal of VSG. Loss of membrane permeability of the dead parasite is illustrated by the dotted line surrounding the parasitophorous vacuole membrane (PVM) and parasite plasma membrane (PPM). Dark red color indicates the emitted fluorescence signal of PI. FSC-H, forward scatter height; SSC-H, side scatter height; VSG, ViSafe Green; PI, propidium iodide; PF, paraformaldehyde; GA, glutaraldehyde; uRBCs, uninfected red blood cells. Counts were determined from two independent readers blinded to the slide identifications. The results are presented as the mean ± SEM of three independent experiments.

We tested methods for enhancing the cell entry of the cell-impermeant fluorophore. Notably, 0.0003% Triton X-100 caused no hemolysis (Fig. 2A) and made the erythrocyte membrane permeable with minimal damage to the parasite membrane compared to 0.001% Triton X-100 (see Supplementary Figure S2 online). Thus, 0.0003% Triton X-100 was subsequently used. To examine the effect of detergents on the viability of the parasites, the synchronized ring-stage parasites were incubated in buffer containing Triton X-100 or paraformaldehyde (PFA)- glutaraldehyde (GA) followed by the culture. Parasitemia of the Triton X-100-exposed parasites increased from the initial 0.5% to 4.8% on day 6, a similar finding to that in the PBS-exposed control experiment. In contrast, the percentage of parasitemia dropped after incubation with PFA and GA (Fig. 2B). After incubation with Triton X-100, the growing trophozoites and schizonts were observed twice during the 6-day culture. The PFA-GA-exposed parasites had condensed nuclei with scant cytoplasm on all examined dates (see Supplementary Figure S2 online). The flow cytometric profiles showed that 3.87 ± 0.16% of the ViSafe Green (VSG)-positive and the propidium iodide (PI)-negative cells was revealed after exposure to 0.0003% Triton X-100, while there were 3.83 ± 0.21% of VSG-positive and PI-positive cells present following incubation with PFA-GA (Fig. 2C). The detected PI signal in the parasitized cells implied loss of membrane integrity of the parasite, consistent with the pyknotic-like morphology observed under a light microscope. Therefore, 0.0003% Triton X-100 did not retard parasite growth and allowed cell-impermeant PI to enter the erythrocytes followed by selective internalization into the Plasmodium membrane to bind DNA of nonviable parasites.

### Increased cell permeability facilitates the use of VSG and PI to fractionate DHA-exposed ring-stage parasites

**Figure 3:**
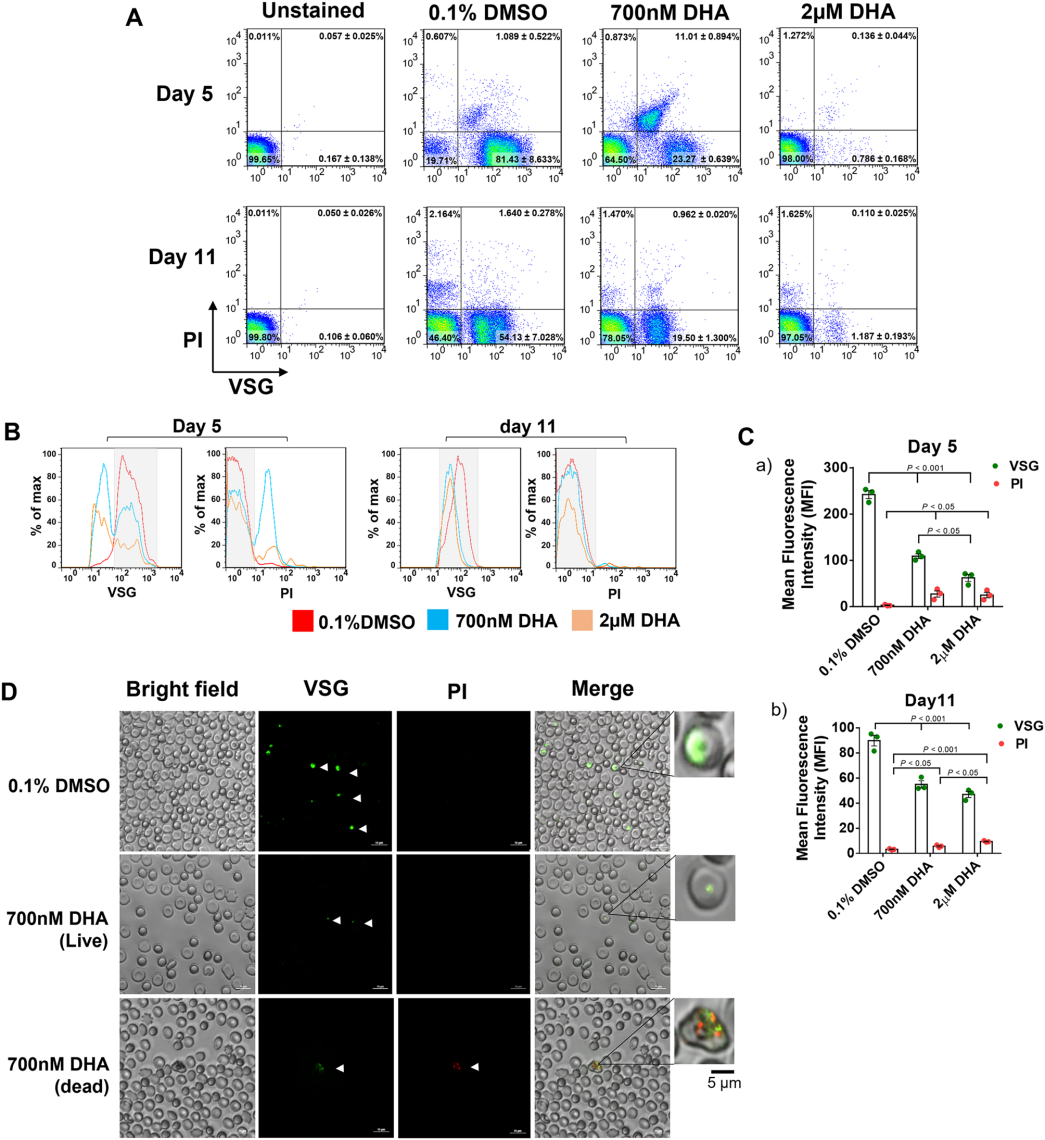
Analysis of DHA-induced dormant and dead parasites using flow cytometry and confocal microscopy. (**A**) Representative flow cytometric profiles of parasites exposed to 0.1% DMSO, 700 nM DHA or 2 µM DHA for 6 h, followed by three daily 5% D-sorbitol treatments, and analyzed on days 5 and 11 post-culture. The erythrocytes were permeated using 0.0003% Triton X-100 and subsequently stained with VSG and PI. After gating FSC-H and SSC-H, cells were sorted based on the fluorescence intensity of VSG and PI. Dead parasites were defined as VSG-positive, PI-positive cells, while dormant parasites were defined as VSG-positive, PI-negative cells. (**B**) Histograms showing the fluorescence intensity of VSG and PI among the DMSO-exposed parasites (red line), 700 nM DHA-exposed parasites (blue line) and 2 µM DHA-exposed parasites (orange line) on days 5 and 11. The viable cells are shown in the grey band. (**C**) Mean fluorescence intensity (MFI) of VSG and PI in the DMSO- and DHA-exposed parasites on days 5 (a) and 11 (b). Each bar represents the MFI in live or dead parasites. The dots on top of each bar represent the three independent experiments. (**D**) Representative confocal images of DHA-induced dormant and dead parasites. Cells are displayed according to the bright field, VSG (V) in green and PI (PI) in red. Higher magnified views of the parasitized erythrocytes are shown in boxes. Scale bar = 5 µm. DHA, dihydroartemisinin; VSG, ViSafe Green; PI, propidium iodide; uRBCs, uninfected red blood cells.

To demonstrate the use of VSG and PI for the analysis of parasitized erythrocytes post antimalarial drug treatment, ring-stage parasites were first exposed to 700 nM or 2 µM DHA and subsequently treated with 5% sorbitol every 24 h for 3 days to select the growth-arrested form of parasites. On days 5 and 11 post exposure, parasitized erythrocytes were isolated from uninfected erythrocytes using the SLOPE method (see Supplementary Figure S3 online). Following gradient centrifugation in Percoll, the cell pellet was incubated with Triton X-100-containing buffer and stained with VSG and PI. In the flow cytometric dot plots, the DHA- and DMSO-exposed parasites are presented based on the fluorescence intensity of cell-permeant VSG and cell-impermeant PI displayed on the X- and Y-axes, respectively (Fig. 3A). On day 5 of 700 nM DHA exposure, the VSG-positive, PI-negative cells located in the lower right quadrant represent the parasites with intact cell membranes, hereby defined as viable (23.27 ± 0.639%). In contrast, the upper right quadrant contains the VSG-positive and PI-positive cells (11.01 ± 0.894%), which indicate that the parasites lost membrane integrity, hereinafter called nonviable parasites. When the ring-stage parasites were exposed to a higher concentration of 2 µM DHA, only 0.786 ± 0.894% of the parasites were viable (lower right quadrant, Fig. 3A). In contrast, a greater number of viable parasites (VSG-positive and PI-negative cells) was found in the DMSO-exposed control experiment (81.43 ± 8.63%). On day 11 of 700 nM DHA exposure, the number of nonviable parasites located in the upper right quadrant (VSG-positive and PI-positive) was reduced to 0.962 ± 0.020%. Regarding the overlaid histogram of the emitted VSG and PI fluorescence, viable cells (grey band) and dead parasites were clearly distinguished after exposure to DHA. There was a slight difference in the maximum fluorescence intensity of VSG between the viable and dead parasites on day 11 of DHA treatment. However, discriminating cell-impermeable staining of DNA among both the viable and dead parasites enabled the reliable identification of viable parasites by PI (Fig. 3B). The mean fluorescence intensity of PI of the DHA-exposed parasites was higher than that of the DMSO-treated control, implying a higher extent of cell membrane integrity loss. Moreover, the significant decrease in the fluorescence intensity of VSG was associated with the growth retardation of DHA exposure (Fig. 3C,  *p *< 0.001). To confirm the ability of the DNA-binding fluorochromes to discriminate between growth-arrested and nonviable parasites, parasitized erythrocytes were observed under a confocal microscope. The growth-arrested parasites were VSG-positive and PI-negative (green-positive cells), while the dead parasites were positive for both VSG and PI (Fig. 3D). Despite binding to DNA, there was no overlapping position between green and red, implying different spatial DNA binding. Overall, cell labeling with a combination of the cell-permeant VSG and the cell-impermeant PI with mild detergent could distinguish between growth-arrested and nonviable DHA-exposed, ring-stage *P. falciparum*.

### The use of VSG and PI does not affect the recrudescence of DHA-induced growth-arrested parasites

**Figure 4:**
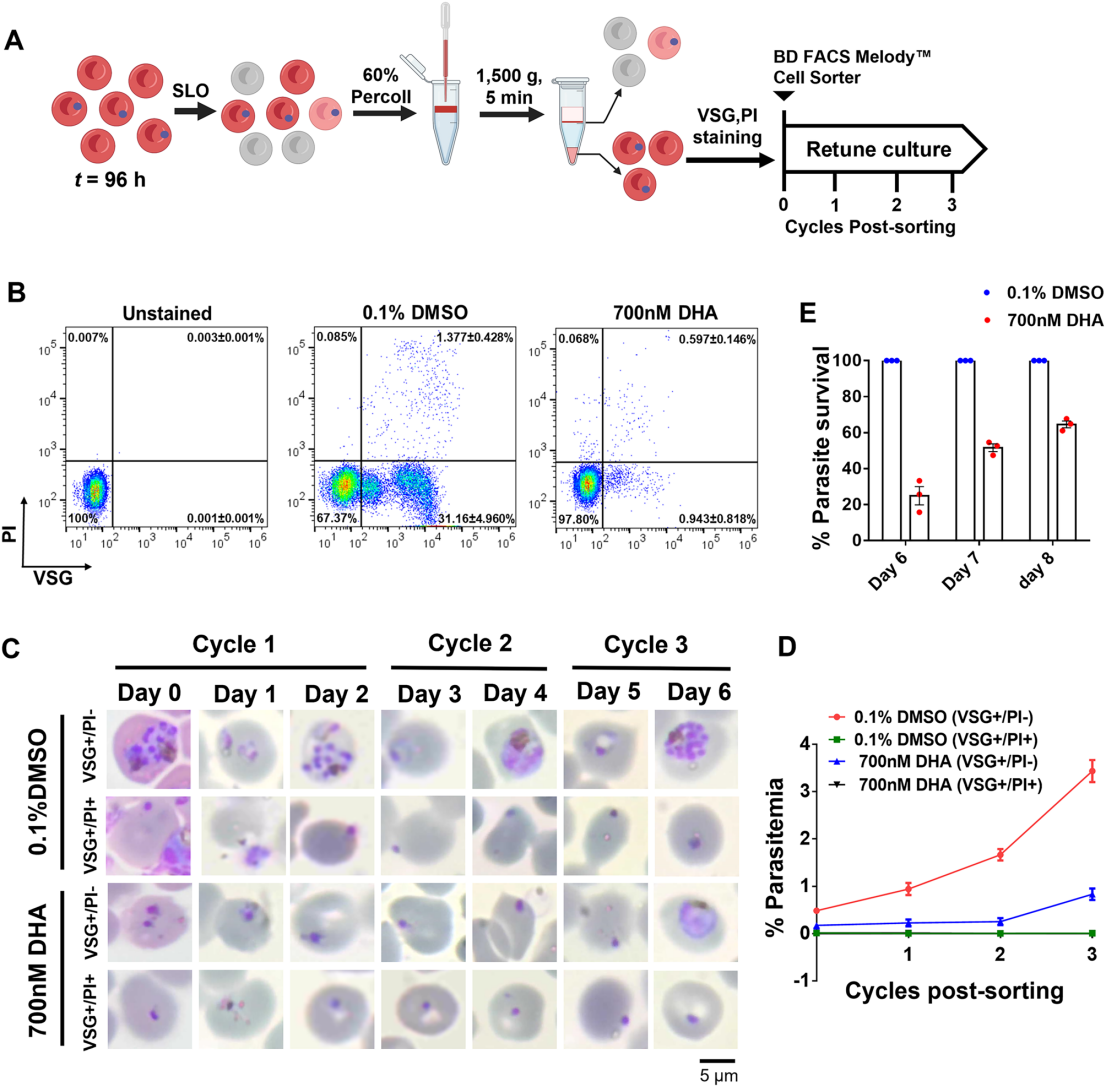
Viability confirmation of dormant *P. falciparum* after DHA exposure. (**A**) Schematic diagram for the separation and culture of DHA-induced dormant parasites. After DHA exposure for 96 h (*t* = 96), the parasites were enriched using the SLOPE method in which the cells were exposed to streptolysin O (SLO) followed by removal of the nonparasitized cells using 60% Percoll. To separate dormant and dead parasites, cells were stained with 10 µg/ml VSG and 10 µg/ml PI, separated into dormant or dead parasites, and cultured for at least three cycles. Parasite growth was monitored using Giemsa-based microscopy. (**B**) Representative flow cytometric profiles for sorting dormant and dead parasites after exposure to 0.1% DMSO or 700 nM DHA. The dormant parasites were VSG-positive and PI-negative cells, while the dead parasites were VSG-positive and PI-positive cells. (**C**) Representative microscopic images of Giemsa-stained erythrocytes show live or dormant cells; VSG-positive, PI-negative and dead cells; VSG-positive, PI-positive. Scale bar = 5 µm. (**D**) Percentages of microscope-based parasitemia after cell sorting and culture for three cycles. (**E**) Parasite survival rate after DHA exposure. By using Giemsa-based microscopy, the percentage of parasites derived from the VSG-positive, PI-negative cells was calculated relative to that of the DMSO-exposed control on days 6 to 8 of the culture. Counts were determined from two independent readers blinded to the slide identifications. The results are presented as the mean ± SEM of three independent experiments.

In RSAs, there is a possibility of DHA-inducted low sensitive stages contributing to false-positive recovery. Thus, we examined the cell morphology in the VSG-positive, PI-negative and VSG-positive, PI-positive fractions on day 4 after exposure to 700 nM DHA for 6 h. A lower percentage of growing trophozoites was observed in the VSG-positive, PI-negative fraction, implying the early recovery of growth-arrested parasites or partial fractions (Supplementary Table S1 online). After DHA exposure and SLOPE treatment, the VSG-positive, PI-negative parasites were sorted and cultured (Fig. 4A and B). In the DMSO treatment group, the parasitemia of the VSG-positive, PI-negative parasites increased over the 6 day period. The VSG-positive, PI-negative parasites obtained after DHA treatment remained dormant for 2–4 days in culture but resumed their growth on day 6 postculture. These data suggest that growth of the DHA-treated parasites was retarded for 48 to 96 h and recrudescence occurred in the third cycle. The VSG-positive, PI-positive populations obtained after exposure to both DMSO and DHA exhibited pyknotic forms that could not develop into subsequent stages (Fig. 4C and D). Moreover, the percentage of parasite survival demonstrated no growth defects of viable parasites with VSG-positive/PI-negative populations after the third cycle (Fig. 4E). Therefore, the use of VSG and PI after drug exposure had no effect on parasite growth.

### Trio fluorochrome assay facilitates the detection of growth-arrested P. falciparum in mock ex vivo RSA

**Figure 5:**
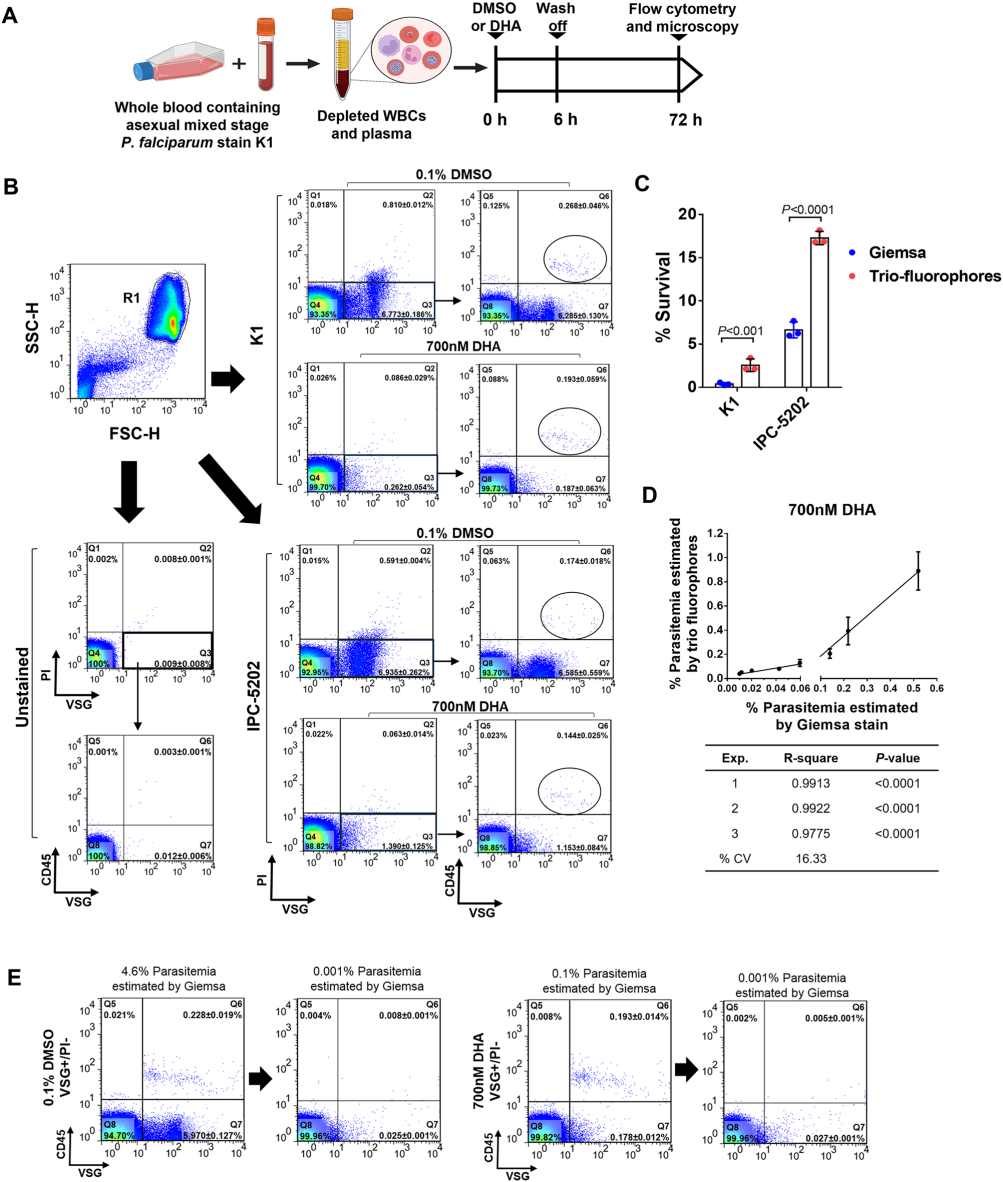
Application of VSG and PI combination-based flow cytometry for the mock ex vivo RSA. (**A**) Schematic diagram of mock ex vivo RSA. To mimic the assay in laboratory settings, mock patient blood samples were prepared by spiking the mixed stages of *P. falciparum* into human blood at a final density of 1% parasitemia. Plasma and white blood cells (WBCs) were removed prior to being spiked with parasites. At the start of the experiment (t = 0 h), nonsynchronized parasite-containing mock blood samples were treated with either 700 nM DHA or 0.1% DMSO for 6 h. After drug removal, the parasites were cultured for 66–72 h followed by analysis using Giemsa-based microscopy and flow cytometry. (**B**) Representative flow cytometric profiles of the cultured parasites 72 h post culture. Given the presence of WBCs in the mock blood, anti-CD45 antibodies were used in the analysis, generating the trio fluorophore assay. Red blood cells and white blood cells were first gated (R1) as FSC-H and SSC-H. Next, only viable cells that were VSG-positive and PI-negative (in Q3) were subjected to profiling based on the fluorescence intensity of CD45 and VSG. The CD45-positive WBCs appeared in gate Q6. The Q6-subtracted viable cells (Q7) and noninfected red blood cells (Q4) were used to calculate parasitemia. (**C**) Bar graph comparing survival percentage for ART-sensitive K1 and ART-resistant IPC-5202 measured using microscopic examination or flow cytometric analysis. (**D**) Correlation between the standard microscopy method and the trio fluorophore assay in the measurement of parasitemia. Spearman’s rank correlation coefficient was used for statistical analysis. The percentage of DHA-induced dormant *P. falciparum* was obtained from flow cytometry (Y-axis) and Giemsa-based microscopy (X-axis). Three independent analyses were performed using a parasitemia range of 0.01–0.5%. (**E**) Sensitivity of the trio fluorochrome assay in the detection of parasitemia compared to that of Giemsa-based microscopy. Flow cytometric profiles of the ex vivo RSA show percentages of CD45-negative, VSG-positive and PI-negative cells.

To apply VSG and PI in a mock ex vivo RSA, simulated patient blood samples containing the laboratory cultured *P. falciparum* ART-sensitive K1 and ART-resistant IPC-5202 strain at different stages and human whole blood were subjected to DHA exposure (Fig. 5A). Given the DNA binding ability of VSG and PI, leukocytes present in the mock blood sample may interfere with the detection of parasitized erythrocytes. A third fluorochrome-labeled antibody specific to the human leukocyte CD45 was added to the combination of VSG and PI, hereafter called the trio fluorochromes. To remove cell debris, cells were first gated based on two laser diffraction methods: forward scatter (FSC) and side scatter, as seen in the pseudocolor dot plots. The cell debris-free population exhibited high FSC and SSC intensities (gate R1 defined as FSC-H and SSC-H, Fig. 5B). The FSC-H and SSC-H populations were then profiled based on the fluorescent signal intensities of VSG and PI (X-axis and Y-axis, Fig. 5B). Two lines were drawn based on the fluorescence intensity of the unstained samples to set a cutoff for positive and negative signals, resulting in quadrants (Q). The VSG‒positive and PI‒negative cells in quadrant no. 3 were viable or growth-arrested parasites. In combination with anti-CD45 antibodies, leukocytes were positive for CD45 and VSG (Gate Q6, Fig. 5B) and excluded from the VSG-positive population. To quantify parasitemia, the number of the VSG‒positive and CD45‒negative population in gate Q7 were calculated as a percentage among the number of cell debris-free populations (gate R1, Fig. 5B). The ratio of viable parasites in the DHA- and DMSO-exposed samples was estimated as the survival percentage. Based on flow cytometric analysis, the range of survival percentage for ART-sensitive parasites was 2 to 3%, whereas ART-resistant parasites ranged from 15 to 18%. Moreover, the survival percentage of ART-sensitive K1 and ART-resistant IPC-5202 calculated from the trio-fluorochromes was significantly higher than those estimated by Giemsa stain (*P* < 0.001 and *P* < 0.0001, respectively) (Fig. 5C). Therefore, the growth-arrested parasites were reliably detected in both ART-sensitive and ART-resistant parasites.

### The performance of the trio fluorochrome assay is comparable to that of the standard microscopy method

To assess the performance of the trio fluorochrome assay, we examined the reliability, reproducibility and sensitivity of the assay in measuring parasitemia. For reliability, different percentages of DHA-induced growth-arrested parasites were added to human blood and subjected to the standard microscopy method and the trio fluorochrome assay. Based on the percentage of cells in quadrant number 7 (Gate Q7, Fig. 5B), parasitemia was detected in a dose-dependent manner using the trio fluorochrome assay. Linear correlation showed that compared to the standard microscopy technique, the trio fluorochrome assay was reliable in measuring parasitemia (n = 3 independent experiments, r^2^ = 0.9775‒0.9913; *p* < 0.0001) (Fig. 5D). The reproducibility of the trio fluorochrome assay was assessed by calculating the coefficient of variation (CV) of three independent experiments. The CV was 16.33%, suggesting low variation between independent experiments.

Next, we compared the limit of detection of the trio fluorochrome assay to that of the standard microscopy. A 0.01% parasitemia solution was prepared and subjected to the trio fluorochrome assay. The results showed that the percentage of cells identified as VSG-positive, PI-negative was higher than that observed using Giemsa-based microscopy (*p* < 0.05, see Supplementary Figure S5 online). To determine the limit of detection in the mock ex vivo RSA, the 72-h cultured parasite suspension (t = 72 h) was diluted to 0.001%. The trio fluorochrome assay detected 0.027 ± 0.001% of the VSG-positive/PI-negative/CD45-negative cells from the sample exposed to DHA, indicating more than 20 times higher sensitivity (Fig. 5E). Thus, the combination of VSG, PI and anti-human CD45 antibodies in a single tube is a reliable, sensitive, and reproducible method for the identification of growth-arrested and nonviable *P. falciparum* in a human blood-like environment.

### Lyophilization of the trio fluorochromes prolongs the stability of the assay and allows cold chain-free transportation

**Figure 6:**
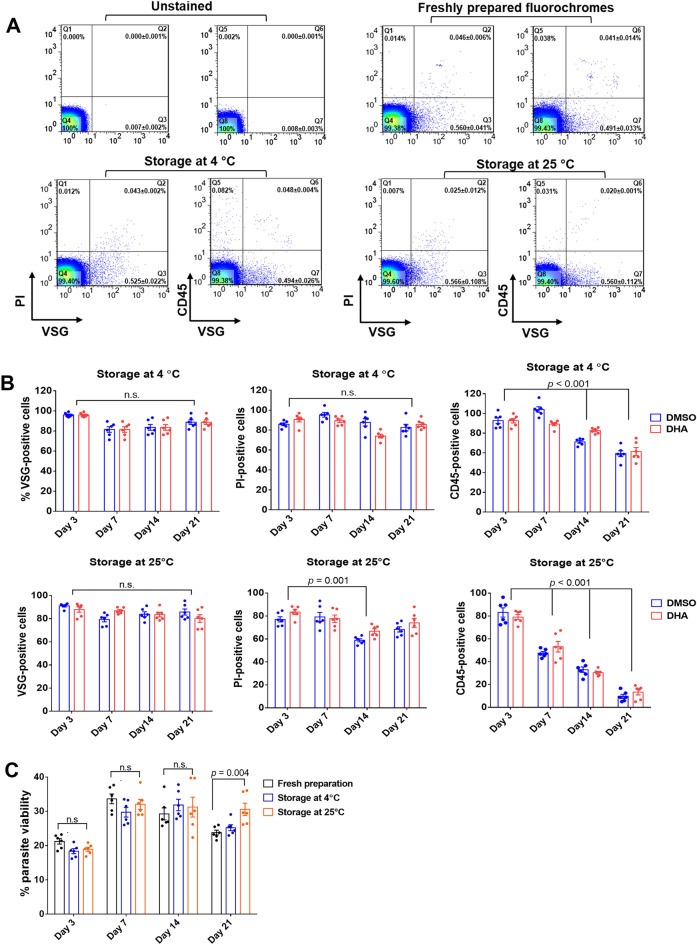
Stability of the lyophilized trio fluorochrome in the detection of DHA-induced dormant *P. falciparum*. (**A**) Flow cytometric profiles of the *P. falciparum* culture at 72 days post DHA exposure. For the trio fluorochrome assay, the lyophilized form was stored at 4 and 25 °C for 7 days before use. Freshly prepared fluorochromes were used for comparison. (**B**) Bar graphs of the percentages of VSG-positive, PI-positive and CD45-positive cells. The trio fluorochromes were stored at different temperatures for 3, 7, 14 and 21 days. Flow cytometric profiles of cells analyzed by using the 4C- or 25C-stored fluorophores are shown in the upper or lower panels, respectively. Blue indicates DMSO-exposed cells, while red indicates DHA-exposed cells. Each dot indicates one of three independent experiments. Data are presented as the means ± SEMs of three independent experiments. (**C**) Bar graph of parasite viability assessed by the trio fluorochrome assay. Viable parasites are defined as VSG-positive, PI-negative and CD45-negative cells and are stained with fluorophores that are stored at different temperatures at different time points. Each dot represents an independent experiment.

Temperature sensitivity limits the use of fluorescence-based assays in resource-restricted areas. Thus, we next assessed the long-term stability of fluorescence probes and antibodies that detect parasitized erythrocytes. The lyophilized forms of VSG, PI and antibody were stored at different temperatures. After 7 days of storage at 4 °C, the flow cytometric profiles showed dot plot patterns similar to the freshly prepared reagent (Fig. 6A). At 4 °C, the lyophilized mixture stably detected parasitized erythrocytes for up to 21 days. However, the lyophilized PI stored at 25 °C lost this ability since there was a significant (30%) decrease in the percentage PI-positive cells after 14 days of storage at 25 °C (*p* = 0.001, Fig. 6B). The lyophilized anti-CD45 antibodies were stable at 25 °C for only 3 days. For the detection of parasite survival, storage of the lyophilized mixture at 4 °C preserved the stability of VSG, PI and anti-CD45 for up to 21 days without any reduction of target binding activity (*P* = 0.004, Fig. 6C). Thus, the lyophilized trio of fluorochromes is stable for up to 14 days at 4 °C.

## Discussion

Observing *P. falciparum* morphology microscopically is a common method that has been readily used for the assessment of antimalarial drug susceptibility in the RSAs. Nevertheless, this microscope-based assay has been impeded by subjective low-throughput processes and is incapable of distinguishing growth-arrested parasites from nonviable parasites. In this study, we applied fluorescence-based flow cytometry to in vitro and mock ex vivo RSAs to examine the effects of artemisinin against the *P. falciparum* K1 strain. We also confirmed that early ring-stage parasites became temporal growth-arrested after DHA exposure in vitro. A key advantage of flow cytometry is the ability to analyze hundreds of thousands of cells within one minute, which is a much faster than microscopic observation. Together with fluorescent signals, this method overcomes the limit of parasite detection of the standard Giemsa-based microscopy method. The use of two fluorophores with different cell permeabilities and an anti-CD45 antibody allows sensitive and specific identification of growth-arrested *P. falciparum* after artemisinin exposure in human blood.

Artemisinin reportedly inhibits the growth of *P. falciparum* in malaria patients for various lengths of time depending on parasite genetic background and drug doses^22^. In the in vitro culture of clinical isolates, the artemisinin-derivative DHA at doses greater than the 90% inhibition concentration failed to completely eliminate the parasites; this delayed clearance likely results from the parasite’s quiescent adaptation^23^. Under a light microscope, growth-arrested *P. falciparum* parasites morphologically resemble nonviable pyknotic cells^24^. Thus, microscopic observation is subjective and dependent on the examiner, leading to inter- and intrareader variation. Therefore, we proposed a morphology-free readout to evaluate drug susceptibility. A single tube containing the three fluorochromes was developed for detecting dead and growth-arrested malaria parasites after artemisinin treatment. This trio fluorochrome assay exhibited high specificity and sensitivity when detecting growth-arrested parasites and may predict artemisinin resistance in a simple and rapid manner. The trio fluorochrome assay overcomes the substantial drawbacks of the standard Giemsa-based microscopy technique. It is completed in a single step, requires a small volume of blood (1–2 µl), and takes approximately twenty minutes to incubate at room temperature without cell fixation and dye washing, allowing routine diagnosis with minimal training and supervision in field settings.

Advances in fluorescence-based cell analysis allow more specific and sensitive detection of organelles and biomolecules of a given cell type, including *Plasmodium* spp^25^. Given the lack of nuclei, the detection of intraerythrocytic parasites primarily relies on the use of any fluorescent probe that binds to the DNA of *P. falciparum*. However, both growth-arrested and dead parasites have *Plasmodium* DNA, increasing false positives in drug tests. Therefore, other non-DNA molecules or nonnuclear compartments should be selected as alternative targets. Mitochondria-binding fluorescent probes have been used for detecting membrane polarity, an indicator of functioning mitochondria in live *P. falciparum*^26,27^. Thus, mitochondrial function has been proposed as a surrogate marker of active and quiescent cells. The combination of a DNA-binding fluorochrome (e.g., SYBR green I), and a mitochondria-binding probe (e.g., MitoTracker Deep Red FM (MTDR)), is able to discriminate growth-arrested parasites (SYBR-positive and MTDR-positive) from pyknotic parasites (SYBR-positive and MTDR-negative)^28^. In addition to MitoTracker, active mitochondria capture the cell-permeant fluorescent probe rhodamine 123. Growth-arrested *Plasmodium* parasites that are positive for rhodamine 123 exhibit delayed regrowth due to the deleterious effect of rhodamine 123 staining^27,29^. Despite the utility of MitoTracker and rhodamine 123, detection of mitochondrial membrane function requires an incubation step at 37 °C as well as a few additional steps to wash out the excess dyes. The high expense of mitochondria-staining dyes also limits their use in low-resource settings. Moreover, ex vivo or in vivo RSAs use early ring-stage parasites. The mitochondrial size is relatively smaller in ring-stage trophozoites than in other intraerythrocytic stages^30^. Therefore, the sensitivity of both mitochondria-detecting fluorophores needs further validation.

In the trio fluorochrome assay, two different types of DNA-binding fluorophores were selected based on (1) the ability to permeate erythrocyte membranes and (2) different emitted fluorescence signals. The *P. falciparum*-infected erythrocytes contain a double-layer cell membrane; one belongs to the erythrocyte and another belongs to the parasite. We hypothesized that the infected erythrocyte at the ring-stage retains a selective cell membrane regardless of the parasite survival status. This hypothesis was supported by the use of the cell impermeant fluorophore PI, which failed to enter the parasitized erythrocytes. In contrast, the cell permanent fluorophore VSG could enter erythrocytes and bind to the *Plasmodium* DNA. We also reasoned that nonviable parasites would lose such selective membrane properties similar to the process of cell apoptosis or necrosis^31^. Therefore, PI could enter nonviable parasites that had a compromised cell membrane. Given the cell impermeability of PI, a way to allow PI to enter erythrocytes before selective permeation through the parasite membrane was needed. Detergents are potent solutions that solubilize the phospholipids of cell membranes. However, too high of a concentration of any detergent causes hemolysis. Here, the optimal concentration of Triton X-100 allowed cell entry of PI without hemolysis or cytotoxicity to the parasites. To prove the concept, we selected cell-permeant and cell-impermeant fluorophores that emit maximum fluorescence signals at different wavelengths and are applicable for most flow cytometers equipped with common lasers. Previously, our group reported the use of the cell-permeant ViSafe green (VSG) to detect *P. falciparum*-infected erythrocytes^21^. Given its common use in cell survival tests, the cell-impermeant PI was selected to combine with VSG. This approach clearly confirmed that the growth-arrested parasites retained an intact membrane (VSG-positive and PI-negative) and resumed growth within 6 days after cell sorting, whereas those that lost the parasite membrane (double-positive for VSG and PI) failed to regrow, confirming the indicator of truly growth-arrested parasites. Therefore, the use of the VSG and PI fluorophores effectively discriminated between growth-arrested and nonviable parasites in an RSA.

Regarding our attempts to permeate erythrocyte membranes, there are several chemical detergents that are widely used in most laboratories. Absolute methanol is usually used for permeabilizing the cell membrane to allow Giemsa staining. However, it is not compatible with many fluorescence-based flow cytometry assays because it causes erythrocyte aggregation^32^. PFA and GA are additional fixative solutions that are used before the PI staining of *Plasmodium* DNA^20^. However, we observed that PFA and GA killed the *Plasmodium* parasites as they decreased parasitemia and impaired parasite growth. Recently, various nonionic detergents, such as poly [oxyethylene (n) nonylphenol], polyoxyethylene alkyl ethers, octyl-β-D glucopyranoside, and Triton X-100, have been utilized for cell membrane permeabilization^33–35^. These nonionic detergents disrupt conductivity and change protein conformation, allowing pores to form on the cell membrane^36^. A low concentration of Triton X-100 was sufficient to permeabilize the erythrocyte membrane. Cell lysis was complete when using Triton X-100 above the critical micelle concentration or 0.22 to 0.24 mM (0.02 wt%)^37^. Here, a low concentration of 0.0003% Triton X-100 was optimal for solubilizing VSG and PI, permeabilized the RBC plasma membranes but not parasitophorous vacuole membrane and parasite plasma membrane. Given the concern regarding the environmental toxicity of the degraded product of Triton X-100, an alternative detergent should be considered for eco-friendly use, such as Nereid, a phenol-free detergent^38^, or Simulsol SL 11 W^39^. Given their commercial unavailability, the use of both of these detergents for the detection of growth-arrested *P. falciparum* needs to be validated.

A limitation of this DNA-binding fluorophore assay is an inability to discriminate between leukocytes and *Plasmodium*-infected erythrocytes. To address this problem, Malleret et al.^40^ used antibodies specific to CD45, a pan surface marker of human leukocytes, together with DNA-binding fluorescence dye to detect *P. falciparum*-infected erythrocytes in blood samples. Thus, we combined the duo fluorophore with third anti-CD45 fluorophore, generating the trio fluorophore assay. Product stability is very important when developing a test kit. To know the product stability after storage for certain periods of time and at different temperatures is beneficial for the transportation and use in field settings. The trio fluorophore assay in a single tube could be stored at a temperature of 4 °C for 14 days. Despite their utility, the stability of antibodies needs to be improved to increase their shelf life. Long-term storage at a higher temperature of 25 °C decreased antibody stability, resulting in a false positive signal due to confounding fluorescence signals emitted from the VSG-binding DNA of human leukocytes. Since antibodies are not thermostable, degradation is expected when exposed to higher temperatures. To extend the shelf life of the products, the use of other fluorophore-labeling molecules for the detection of leukocytes, such as smaller and more stable forms of CD45-binding single chain variable fragments, nanobodies or even peptides, may be useful^41^.

The artemisinin sensitivity of *P. falciparum* depends on intraerythrocytic growth stage. The early ring-stage parasites that invade erythrocytes for 2–4 h are hypersensitive to artemisinin, while the ring-trophozoite transition stage (8–20 h -post invasion) has lower sensitivity to artemisinin relative to the early ring form^42^. To ensure nearly complete removal of the lowly DHA-sensitive stages prior to the trio fluorochrome assay, three rounds of D-sorbitol treatments were performed to exclude the trophozoites that were not affected by the DHA. Additionally, these trophozoites with low sensitivity to DHA are capable of rapidly recovering from drug exposure. Hence, these three consecutive D-sorbitol treatments also eliminated these forms with low sensitivity to DHA. Given the lack of definitive markers of growth arrest, this study was able to confirm growth-arrested ring stage *P. falciparum*. However, the existence of the growth-arrested intraerythrocytic stage remains debatable. Recovery of the parasites after artemisinin treatment originates from residual parasite below the limit of detection. Debating the existence of true growth arrest is out of the scope of this study, which aimed to apply a novel trio fluorochrome method to the RSA. As we extended the findings from the previous report, SLO and Percoll were used for the purification of growth-arrested parasites. This report may allow further discovery of other biological markers of cell growth arrest. In conclusion, the assay proposed here is a fast, accurate and versatile method for detecting artemisinin-induced growth-arrested parasites. This method can be further optimized for use with a low-cost, 2 laser-equipped flow cytometer, high-content fluorescence imager^43^ or an on-chip cell analyzer^44^, thus promoting the prevention and surveillance of antimalarial drug resistance in low resource settings or high-throughput drug screening.

## Materials and methods

### Plasmodium falciparum culture

The *P. falciparum* parasite K1 strain was routinely maintained in culture as previously described^45^. Briefly, the parasites were cultured with human erythrocytes at 5% hematocrit. Donors who had blood group O were recruited for venous blood collection. Malaria culture media (MCM) consisted of RPMI 1640 (HyClone, Marlborough), 25 mM HEPES (Gibco, Nanyang, Singapore), 0.2% NaHCO_3_, and 10% (vol/vol) heat-inactivated human serum obtained from blood group B individuals. The parasites were incubated at 37 °C in a 5% CO_2_ and the medium was changed every two days. Parasite growth was monitored by the morphological observation of Giemsa-stained blood smears using a light microscope.

### Ethics statement

Ethical approval for this study was provided by the Human Research Ethics Committee of the Faculty of Medicine, Siriraj Hospital, Mahidol University (COA 408/2023). Experiments involving human erythrocytes and serum obtained from the healthy human participants were performed in according with relevant guidelines and regulation. All samples were handled in accordance with approved protocols and carried out in agreement with ethical standards of the Declaration of Helsinki. The bloods were drawn after written informed consent from all participants in accordance with approval by the Human Research Ethics Committee of the Faculty of Medicine, Siriraj Hospital, Mahidol University.

### Giemsa staining and microscopy

To prepare a thin blood smear, the cultured erythrocytes were spread on a glass slide and absolute methanol was added to fix the cells. The slides were incubated with a 1:18 diluted Giemsa solution at 25 °C for 30 min. After staining, the glass slides were washed with running tap water, air-dried, and mounted with a cover glass using Permount mounting solution. Cell morphologies were observed under a light microscope (Olympus BX53) using 100 × objective lens with oil immersion. To calculate parasitemia, a minimum of 1,000 erythrocytes were examined by two independent microscopists. The intraerythrocytic stages of *P. falciparum* were determined according to the previous research of Silamut et al.^46^.

### Ring-stage survival assay

Resistance to dihydroartemisinin (DHA) was examined using a ring-stage survival assay (RSA) in which the presence of ring-stage parasites was examined 3 h after merozoites invaded erythrocytes (known as RSA^0–3 h^). The in vitro and ex vivo RSAs were performed following a previously described protocol with minimal modification^9^. For the in vitro RSA^0–3 h^, the synchronized ring-stage parasites were prepared by performing two rounds of treatment with 5% D-sorbitol followed by cell culture for 44 to 48 h, allowing parasite development into schizonts containing 10–12 nuclei. Then, the schizonts were isolated using 60% Percoll and subsequently mixed with 200 µl of blood group O erythrocytes (50% hematocrit). The isolated schizonts were cultured with freshly prepared blood group O erythrocytes for 3 h followed by synchronization using 5 volumes of 5% D-sorbitol to obtain early ring-stage. For drug exposure, the early ring-stage parasites were diluted to 3% parasitemia and prepared in 2.5% hematocrit at a final volume of 2 mL. The ring-stage parasites were exposed to 700 nM and 2 µM DHA and incubated in a 5% CO_2_ atmosphere at 37 °C for 6 h. Parasites cultured with 0.1% DMSO were used as a negative control. After 6 h of drug exposure, the parasites were washed with RPMI 1640 by centrifugation at 2,500 rpm for 5 min and resuspended in MCM. Following DHA removal, the parasites were cultured for an additional 72 h. Viable parasites were detected using flow cytometry and Giemsa-based microscopy. The percentage of parasite survival was calculated using the following formula.

$$\% {\text{parasite}}\,{\text{survival}} = \frac{{{\text{Number}}\, {\text{of}}\,{\text{live}}\,{\text{parasites}}\,{\text{treated}}\,{\text{with}}\,{\text{DHA}}}}{{{\text{Number}}\, {\text{of}}\,{\text{live}}\,{\text{parasites}}\,{\text{treated}}\,{\text{with}}\,{\text{DMSO}}}} \times 100$$

For the mock ex vivo RSA, simulated patient blood was prepared by mixing all stages of the *P. falciparum* ART-sensitive K1 or ART-resistant IPC-5202 strain (BEI Resources, NIAID, NIH, USA) with whole venous blood. The plasma and leukocytes were removed prior to carrying out drug exposure. Briefly, the intraerythrocytic stages of *P. falciparum* were not synchronized and adjusted to 1% parasitemia and 2.5% hematocrit. The parasites were exposed to either 700 nM DHA or 0.1% DMSO for 6 h, washed, and then cultivated for 72 h. The readout of viable parasites was quantitatively measured using flow cytometry and Giemsa-based microscopy.

### Recrudescence assay

Following 6 h of exposure of the ring-stage parasites (Ring^0–3 h^) to 700 nM and 2 µM DHA, the parasites were treated with 5% D-sorbitol for 20 min at 37 °C and washed twice with MCM at 24, 48 and 72 h postdrug treatment to remove the mature parasites. On days 5 and 11, the cells were identified as growth arrested or pyknotic using flow cytometry and confocal microscopy. Recrudescence of the DHA-exposed parasites was assessed based on microscopic morphology of the Giemsa-stained blood smears. The percentage of viable parasites was calculated after every 24 h of culture. The medium was changed every two days, and fresh erythrocytes were added every 4 days until the parasites resumed their growth.

### Hemolytic activity

To determine the minimum concentration of Triton X-100 at which erythrocytes resist lysis, a hemolytic assay was performed as previously described with modifications^47^. Briefly, human blood group O erythrocytes were diluted with 1 × phosphate buffered saline (PBS) to obtain 3% hematocrit. Fifty microliters of the diluted blood was transferred to a flat-bottom 96-well polystyrene plate and incubated with 50 µl of 0.2–0.00006% (v/v) Triton X-100 for 30 min at 25 °C, resulting in final concentrations of 0.1, 0.01, 0.003, 0.001, 0.0003, 0.0001 and 0.00003%. After incubation, the samples were centrifuged at 2,500 rpm for 5 min. The supernatant was subjected to measuring absorbance (A) at 412 nm using the Synergy™ 118 H1 Hybrid Multi-Mode Reader (BioTek, Winooski, VT). The erythrocyte suspension incubated with 1 × PBS or 0.1% Triton X-100 served as negative and positive hemolysis controls, respectively. The percentage of hemolysis was calculated using the following formula.

$$\mathrm{\% Hemolysis }=\frac{\mathrm{A }({\text{test}}) -\mathrm{A }\left(\mathrm{negative\,control}\right)}{\mathrm{A }\left(\mathrm{positive\,control}\right)-\mathrm{A }(\mathrm{negative\,control})}\times 100$$

### Hemolytic activity of streptolysin O (SLO)

SLO was used for the purification of *P. falciparum* ring-stage parasites^48^. This study adapted the SLO protocol for the purification of growth-arrested *P. falciparum*. To determine the hemolytic unit of SLO optimal for isolating the growth-arrested *P. falciparum*, SLO lyophilized powder (Abcam, Cambridge, UK) was solubilized in 5 ml of activation solution (1 × PBS, 0.1% BSA and 5 mM dithiothreitol) and incubated at 37 °C for 2 h. A 50 µl blood group O erythrocyte suspension at 3% hematocrit was incubated with the SLO solution at different volumes of 0, 0.0625, 0.125, 0.25, 0.5, 1.0 and 1.5 µl at 37 °C for 30 min. After centrifugation, 10 µl of the supernatant obtained from SLO-exposed erythrocyte samples was mixed with 990 µl of 1 × PBS to inhibit cell lysis activity. The SLO-treated samples were transferred to a new 96-well plate, and the absorbance was measured at 412 nm. The percentage of hemolysis was calculated as mentioned above. The number of SLO units that caused 50% hemolysis was identified. The dose‒response curve of SLO concentration and absorbance was plotted using GraphPad Prism v6.

### Effect of detergents on parasite viability

To determine the procedure for the permeabilization of erythrocytes without damaging intraerythrocytic *P. falciparum*, the viability of ring-stage parasites was examined following exposure to detergent and fixative agents. Synchronized ring-stage parasites at 0.5% parasitemia were incubated with three different solutions: 1) 1 × PBS, 2) 0.0003% Triton X-100 prepared in 1 × PBS or 3) 0.5% paraformaldehyde (PFA) and 0.25% glutaraldehyde (GA). Treatment was performed by incubating the synchronized ring-stage parasites with 1 × PBS or Triton X-100 for 30 min and with PFA for 2 h at 25 °C. The PFA-exposed cells were subsequently incubated with 0.25% GA solution for 10 min at 25 °C. After incubation, the cell suspension was centrifuged at 2,500 rpm for 5 min to remove the excess reagent in the supernatant. The cell pellets were then resuspended in 1 mL MCM, plated into a 12-well plate, and incubated at 37 °C and 5% CO_2_ for 6 days. The MCM was renewed every day. The parasitized erythrocytes were monitored every 24 h using a light microscope to observe the Giemsa-stained thin blood smears (see Supplementary Figure S2 online). On day 6, the viability of the parasites was measured using a flow cytometer.

### Enrichment of growth-arrested ring-stage parasites using SLO and Percoll

Given the selective hemolysis of noninfected erythrocytes, a combination of SLO and Percoll (SLOPE) was able to purify ring-stage parasites^48^. This study used SLOPE to purify growth-arrested ring-stage parasites. Briefly, 50 µL of the parasite culture with 3% hematocrit was mixed with 1 × PBS to obtain a final volume of 500 µL. Subsequently, forty units of SLO (see Supplementary Figure S3 online) was added to the cell suspension. The mixture was incubated at 25 °C for exactly 6 min followed by the addition of 5–10 × the total volume of 1 × PBS to stop cell lysis. The mixture was centrifuged at 2,500 rpm for 3 min. After removing the supernatant, the cell pellet was resuspended in 100 µl of RPMI 1640 and then slowly laid on top of 500 µL of 60% Percoll in a 1.5-mL tube. The cell-overlaid Percoll was centrifuged at 3,500 rpm for 5 min at 25 °C. The lysed cell-containing upper layer and the Percoll were removed carefully. The cell pellet was washed once with 1 × PBS. Cell morphology was assessed using Giemsa staining, and the purity of the parasitized cells was calculated using the following formula.

$$\mathrm{\% purity }=\frac{\mathrm{Number\,of\,cells\,with\,parasite\,infection}}{\mathrm{Number\,of\,total\,cellscounted }}\times 100$$

### Fluorescence labeling

DHA-exposed ring-stage parasites at 2.5% hematocrit were centrifuged to prepare the erythrocyte pellet. The erythrocyte pellet (1–2 µL) was resuspended in 100 µL of staining solution consisting of 0.0003% Triton X-100, 10 µg/mL ViSafe green (Vivantis Technologies, Selangor, Malaysia) and 10 µg/mL propidium iodide (Thermo Fisher Scientific, Eugene, OR). To detect growth-arrested parasites in the simulated human blood samples, the staining solution contained an anti-CD45 antibody tagged with allophycocyanin (APC) to distinguish leukocytes (1:50 dilution or 1.25 µg/mL from BioLegend, San Diego, CA). After incubating at 25 °C for 20–30 min with light protection, the excess dye-containing supernatant was removed by centrifugation at 2,500 rpm for 3 min. The cell pellets were resuspended in 300 µl of 1 × PBS containing 0.5% fetal bovine serum, herein called PBS-FBS. The samples were subjected to fluorescence analysis within 30 min using a flow cytometer and confocal microscope. To prepare the cell sample for the fluorescence assays, the mixed-stage *P. falciparum* parasites were exposed to 0.5% PFA and 0.25% GA to increase cell permeability followed by incubation with VSG or PI separately.

### Flow cytometry

The fluorophore-labeled cells were analyzed using a FACSCalibur (BD Biosciences, San Jose, CA) equipped with 488- and 633-nm lasers. The emitted fluorescence of VSG and PI passed through 530 ± 30-nm and 585 ± 42-nm filters, respectively, for detection. For APC-conjugated anti-human CD45, a 661 ± 16-nm filter was used. A total of 250,000 cells flowed into the fluidic channel to be activated by the lasers using CellQuest™ software. The cell suspension stained with a single dye was used to identify the overlapping detection of each fluorophore by other detectors (see Supplementary Figure S4 online). The flow cytometric data were analyzed using FlowJo version 7.6 software (Tree Star, Inc., Ashland, OR). The analyzed cells were displayed based on forward scatter (FSC) and side scatter of fluorescence intensity on a logarithmic scale. To remove cell debris, the cell population was gated according to the low intensity of FSC (FSC^low^) and SSC (SSC^low^). For cell sorting, the SLOPE protocol was used to remove the uninfected erythrocytes, increasing the purity of the sorted live and dead parasites. The SLOPE-enriched cells were resuspended in 1 mL of PBS-FBS and subjected to cell sorting using a BD FACS Melody cell sorter with a 70-micron nozzle. Cell gating was performed using BD FACSChorus™ Software (BD Biosciences, Franklin Lakes, NJ). The stained cells were fractionated into two populations, live cells (VSG-positive, PI- negative) and dead cells (VSG-positive, PI-positive), and sorted into a 1.5-mL tube containing 1 × PBS and 2% FBS. A thin blood smear was prepared for staining with Giemsa dye. To confirm parasite viability after cell sorting, the sorted cells were cultured to monitor their growth. After centrifugation at 2,500 rpm for 5 min, the sorted cell pellet was resuspended in 180 μl of MCM and transferred to a well of a 96-well plate. Twenty microliters of 50% packed erythrocytes were added to each well to obtain a 5% hematocrit at the final concentration. The MCM was changed on alternate days. To replenish lost erythrocytes during cell sampling, 150 μL of erythrocyte suspension (3% hematocrit) was added every 4 or 5 days. Parasitemia was monitored by microscopic observation of Giemsa-stained blood smears.

### Confocal microscopy

To confirm the spatial staining pattern of VSG and PI in the intraerythrocytic *P. falciparum*, 100 μL of the VSG- and PI-stained cells were diluted with PBS containing 0.5% FBS to obtain a final concentration of 1% hematocrit, followed by addition into a flat bottom well of a 96-well plate. The cells were observed under an AX confocal-based superresolution microscope (Nikon Corporation, Tokyo, Japan) equipped with a 20 × objective lens. Differential interference contrast with 488-nm and 633-nm argon-ion lasers was used for visualizing the fluorescent signals. Digital microscopic images were captured using Nikon’s NIS-Elements software.

### Reliability and sensitivity

To assess the reliability of our fluorescence assay, percentages of parasitemia obtained from the fluorescence-based flow cytometric assay were compared to those obtained from the standard Giemsa-based microscopy method. Different levels of parasitemia were prepared by diluting the parasitized erythrocytes with uninfected erythrocytes to obtain parasitemia ranking from 0.01 to 0.5% at a final 5% hematocrit. In the flow cytometry analysis, viable or growth- arrested *P. falciparum* parasites were defined as VSG-positive, PI-negative and CD45-negative cells. Percentages of fluorescence-based parasitemia were calculated using the following formula.

$${\text{\% Parasitemia}} = \frac{{{\text{number}}\,{\text{of }}\,{\text{VSG}} + ,\,{\text{PI}} - \,{\text{and }}\,{\text{CD}}45 - \,{\text{cells }}}}{{{\text{number}}\,{\text{ of}}\,{\text{ total}}\,{\text{erythrocytes}}}} \times 100$$

The correlation between the Giemsa-based standard microscopy method and the fluorescence-based flow cytometry method was estimated using Spearman’s rank correlation coefficient. To evaluate the sensitivity, the *P. falciparum*-infected erythrocytes were diluted to 0.001% parasitemia, which is below the limit of detection in a routine microscopic diagnosis^49^. Then, the samples were stained with VSG, PI and APC-conjugated anti-CD45 antibody and analyzed using flow cytometry as described above.

### Stability test

To determine the stability of the fluorophore-based assay, a mixture of fluorophores was lyophilized and then stored at different temperatures for several days before use. Briefly, 5 µL of 10 µg/mL VSG, 10 µg/mL PI and 1.25 µg/mL APC-conjugated anti-human CD45 were mixed in 1.5-mL light-safe centrifuge tubes. The mixture was rapidly frozen in liquid nitrogen and lyophilized using a freeze dryer (Martin Christ Alpha 1–2 LD plus, Martin Christ Gefriertrochnungsanlagen GmbH, Germany) for 15 min. For the intervariability test, the lyophilized fluorophores and the cell permeable buffer (0.0003% Triton-X100 in 1 × PBS) were prepared for three independent sets, and each set contained duplicate samples (total sample number, n = 6). The fluorophore mixtures were stored at 2–8 °C and ambient temperatures for 3, 7, 14 and 21 days. The active, growth-arrested and dead parasites were detected with the lyophilized mixture after 72 h of DHA exposure. The lyophilized fluorophores were reconstituted in 100 µL of the cell permeable buffer. Subsequently, the reconstituted fluorophore mixtures were mixed with 1–2 µL of the DHA-exposed parasites (50% hematocrit). The stability of the fluorophore mixtures was determined by measuring the percentage of fluorescence-positive cells and parasite survival and comparing them to those obtained from using fresh preparations of the fluorescence dyes.

### Statistical analysis

Data analysis and graph generation were performed using GraphPad Prism software version 6.0 (GraphPad Software, Inc., San Diego, CA, USA). The results are expressed as the mean ± standard error of the mean. Spearman’s rank correlation coefficient was used to measure the strength of the association between standard microscopy and fluorochrome-based flow cytometry. Statistically significant differences were identified using a nonparametric Student’s t test. A *p* value less than 0.05 was regarded as statistically significant.

### Supplementary Information

Supplementary Information.
