# Author Correction: Trio fluorophore-based phenotypic assay for the detection of artemisinin-induced growth-arrested *Plasmodium falciparum* in human erythrocytes

**DOI:** 10.1038/s41598-024-61092-5

**Published:** 2024-05-14

**Authors:** Porntida Kobpornchai, Mallika Imwong, Kasem Kulkeaw

**Affiliations:** 1 Siriraj Integrative Center for Neglected Parasitic Diseases, Department of Parasitology, Faculty of Medicine Siriraj Hospital, Mahidol University, Bangkok, 10700 Thailand; 2 Siriraj-Long Read Lab, Department of Bioinformatics, Faculty of Medicine Siriraj Hospital, Mahidol University, Bangkok, 10700 Thailand; 3 Department of Molecular Tropical Medicine and Genetics, Faculty of Tropical Medicine, Mahidol University, Bangkok, 10700 Thailand

Correction to: *Scientific Reports* 10.1038/s41598-024-52414-8, published online 20 January 2024

The acknowledgements section in the original version of this article was incomplete.

“This research project was supported by Mahidol University and funded by the National Research Council of Thailand (NRCT). We thank the following supporters. For providing the strain of *P. falciparum*, thanks to Associate Professor Dr. Duangdao Palasuwan from the Oxidation in Red Cell Disorders Research Unit, Department of Clinical Microscopy, Faculty of Allied Health Sciences, Chulalongkorn University, and Colonel Professor Dr. Mathirut Mungthin from the Department of Parasitology, Phramongkutklao College of Medicine. Staff at Department of Parasitology, Faculty of Medicine Siriraj Hospital, Mahidol University. For instrumentation support of the AX cronfocal-based superresolution microscope and imaging technique advice, we thank Hollywood International Ltd., and the staff members Arisasa Issarachaiwong. We also thank the Mahidol University-Frontier Research Facility (MU-FRF) and MU-FRF scientists Nawapol Udpuay and Pornphawit Momai for their kind assistance with cell sorting.”

now reads:

“This research project was supported by Mahidol University and funded by the National Research Council of Thailand (NRCT). We thank the following supporters. For providing the strain of *P. falciparum*, thanks to Associate Professor Dr. Duangdao Palasuwan from the Oxidation in Red Cell Disorders Research Unit, Department of Clinical Microscopy, Faculty of Allied Health Sciences, Chulalongkorn University, and Colonel Professor Dr. Mathirut Mungthin from the Department of Parasitology, Phramongkutklao College of Medicine. Staff at Department of Parasitology, Faculty of Medicine Siriraj Hospital, Mahidol University. For instrumentation support of the AX cronfocal-based superresolution microscope and imaging technique advice, we thank Hollywood International Ltd., and the staff members Arisasa Issarachaiwong. We also thank the Mahidol University-Frontier Research Facility (MU-FRF) and MU-FRF scientists Nawapol Udpuay and Pornphawit Momai for their kind assistance with cell sorting. This research project is supported by Mahidol University (Strategic Research Fund: fiscal year 2023).”

The original Article has been corrected.

