# Supplementary material for: Trio fluorophore-based phenotypic assay for the detection of artemisinin-induced growth-arrested Plasmodium falciparum in human erythrocytes
Source: Sci Rep. 2024 Jan 20;14:1802. doi: 10.1038/s41598-024-52414-8 (PMC10799909; doi:10.1038/s41598-024-52414-8)
Supplement: Supplementary file 1 — Supplementary Information. [file 41598_2024_52414_MOESM1_ESM.pdf]

Supplemental information

Trio fluorophore-based phenotypic assay for the detection of artemisinin-induced growth-arrested *Plasmodium falciparum* in human erythrocytes

Porntida Kobpornchai, Mulika Imwong, Kasem Kulkeaw

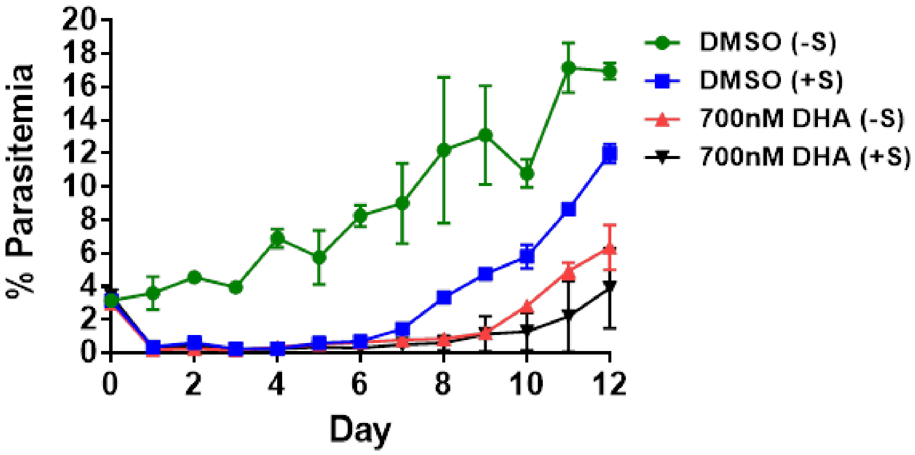

Figure S1. The impact of sorbitol treatment on the recrudescence associated with artemisinin inducted growth- arrested parasite. The DMSO or DHA exposed parasites received the three times of sorbitol treatment (+S) at 24, 48 and 72 h or were non treatment sorbitol (-S). The trophozoite and schizont parasites were lysed by sorbitol, leading to reduction in parasitemia of DMSO-expose parasites (at day 6, % parasitemia = 10% in DMSO (S−) versus 0.8% in DMSO (S+)). In the DHA-treated parasites, there was no impact on the recrudescence curve (at day 10, parasitemia = 2.85% in DHA (S−) versus 1.3% in DHA (S+)).

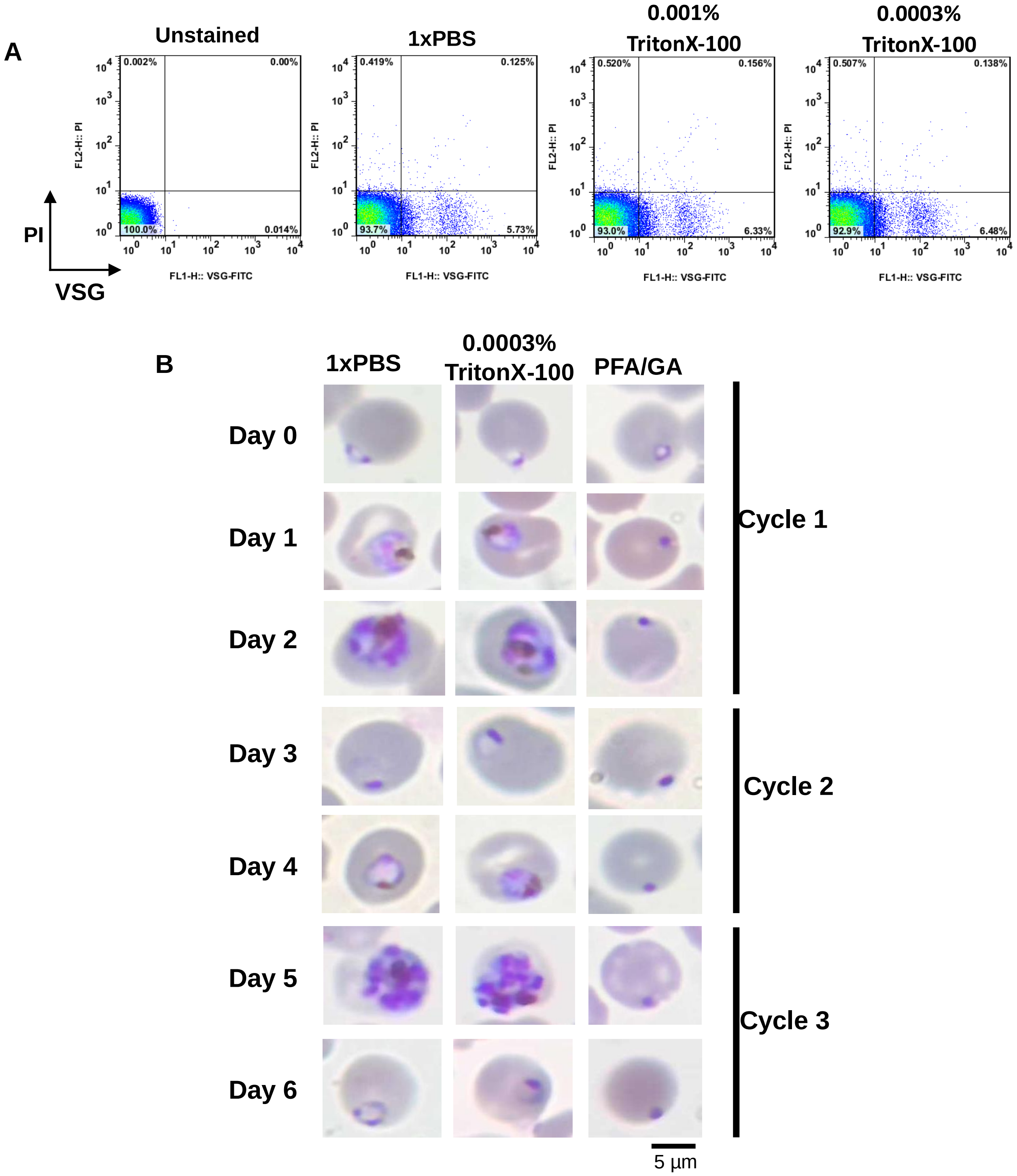

Figure S2. Effect of Triton X-100 on the viability of *P. falciparum* stain K1. A. Dot plot of the representative flow cytometry analysis of the parasites exposed to 0.001% or 0.0003% Triton X-100 for 30 min and subsequently stained with 10 µg/mL VSG and 10 µg/mL PI before analyzing the fluorescent signal. **B.** Representative morphology of the parasites after treatment with three different detergents and additional culture for 6 days. The percentage of parasitemia did not increase, and no other intraerythrocytic stages were observed under a light microscope. Scale bar = 5 µm.

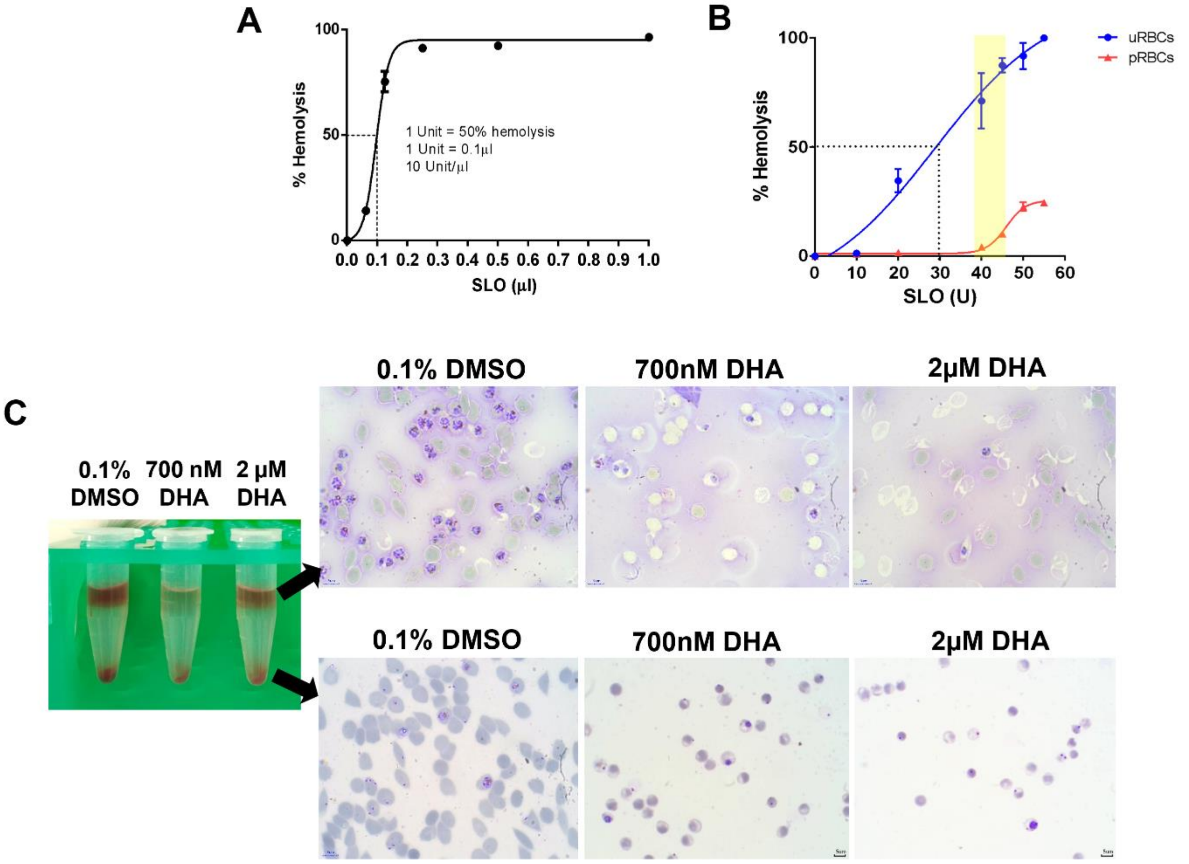

Figure S3. **The enrichment of intraerythrocytic dormant stage parasites using the streptolysin O-Percoll (SLOPE) method. A.** The amount of streptolysin-O (SLO) for one unit which presents 50% lysis of uninfected red blood cells. **B.** Optimal hemolysis unit of SLO for isolation parasite infected red blood cells (pRBCs) from uninfected red blood cells (uRBCs). Dot line were 50% lysis of uRBCs. The yellow band indicated the optimal unit (40U) which lysed the uRBCs approximately 90%. Results are presented as mean ± SEM of three independent experiments. **C.** The gradient centrifugation with 60% Percoll of intraerythrocytic parasites after 0.1% DMSO, 700nM DHA or 2µM DHA exposure. Cell pellets and upper layer of Percoll were visualized by Giemsa stain. Scale bar = 5 µm.

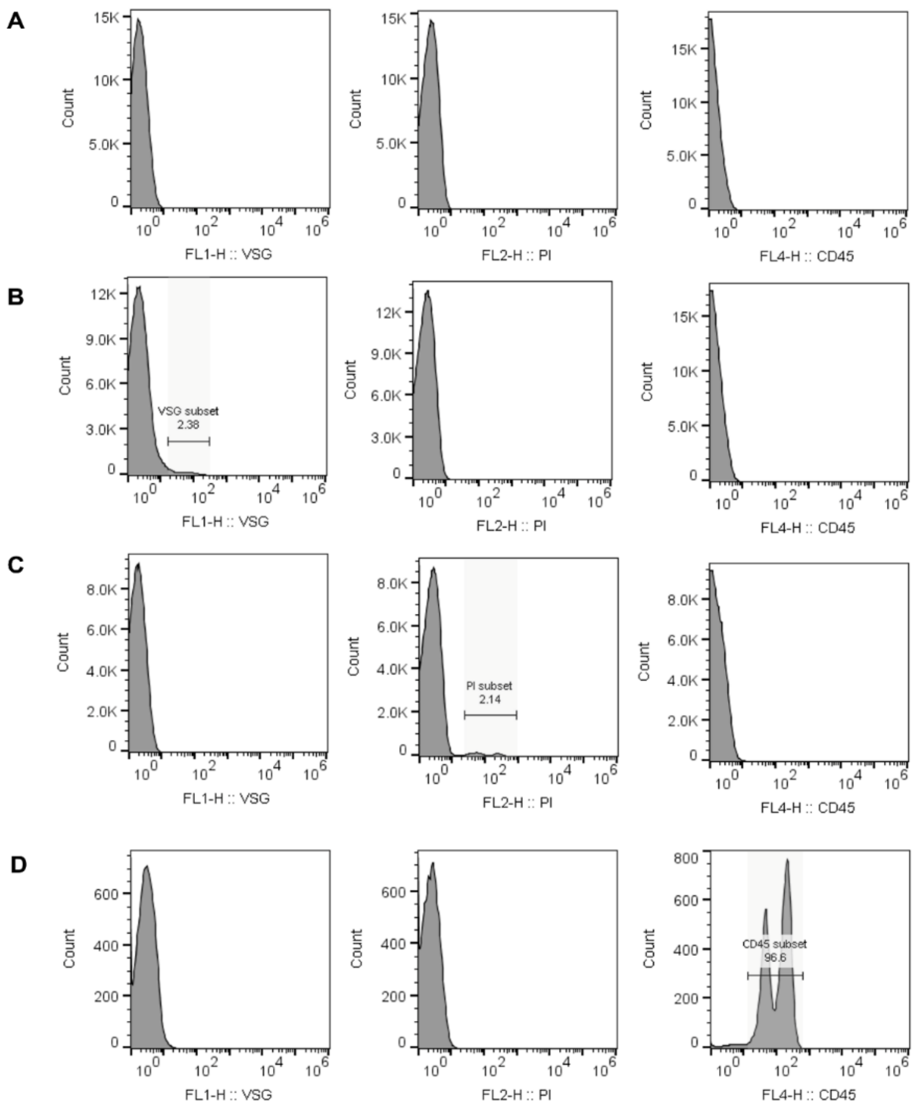

Figure S4. Histogram profile of the single staining cells for compensation setting. A. unstained intraerythrocytic parasites **B.** the non-synchronized intraerythrocytic parasites stained with VSG **C.** the non-synchronized intraerythrocytic parasites treated with 2.5%PFA and 0.5% GA then stained PI. **D.** The peripheral blood mononuclear cells stained with APC anti-CD45.

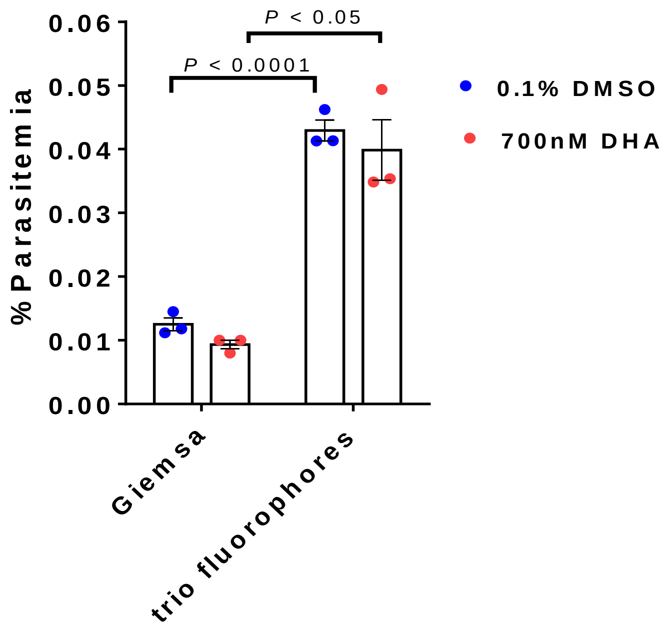

Figure S5. Bar graph comparing parasitemia measured using microscopic examination or flow cytometric analysis. The percentages of VSG-positive, PI-negative, and CD45-negative cells detected using flow cytometry and parasitemia obtained from the Giemsa-based microscopy method are plotted on the bar graph. Three independent settings of *P. falciparum* culture were diluted to 0.01% parasitemia. Each dot represents one of three independent experiments.

Table S1. The summary data of sorting the dormant or dead parasites after exposure to 0.1% DMSO or 700nM DHA.

**Exposure**	**Population**	**Predominant morphology**	**Sort Count (Replicated 1)**	**Sort Count (Replicated 2)**	**Sort Count (Replicated 3)**
0.1% DMSO	VSG+/PI-	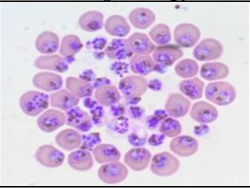	130,000	230,000	288.000
	VSG+/PI+	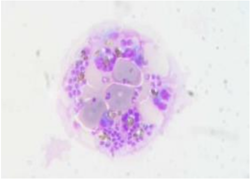	15,000	12,509	30,492
700 nM DHA	VSG+/PI-	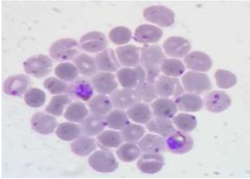	30,000	25,256	35,408
	VSG+/PI+	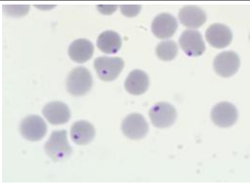	5,000	5,203	2,369
